# Quality of evidence supporting the role of probiotics for rheumatoid arthritis: an overview of systematic reviews

**DOI:** 10.3389/fimmu.2024.1397716

**Published:** 2024-05-30

**Authors:** Weiqing Li, Yalan Zhang, Dandan Guo, Rui Gong, Jiaxin Yuan, Huijun Yang

**Affiliations:** ^1^ Gansu Provincial Hospital of TCM, Lanzhou, China; ^2^ The Second Affiliated Hospital of Fujian Medical University, Quanzhou, China; ^3^ The First Veterans Hospital of Sichuan Province, Chengdu, China; ^4^ Tianjin University of Traditional Chinese Medicine, Tianjin, China

**Keywords:** rheumatoid arthritis, probiotics, evidence, treatment, adjunctive therapy

## Abstract

**Background:**

To evaluate the methodological quality, report quality, and evidence quality of meta-analysis (MA) and systematic review (SR) on the efficacy of probiotics in the treatment of rheumatoid arthritis (RA).

**Methods:**

Databases were used to identify eligible SRs/MAs until February 12, 2024. The methodological quality of the studies was assessed using AMSTAR-2 tool, the quality of the literature reports was scored using PRISMA checklists, and the quality of the evidence was graded using GRADE system.

**Results:**

Seven reviews including 21 outcomes were included. Methodological quality of the included reviews was of general low, and the entries with poor scores were 2, 4, and 7. By PRISMA checklists, there were some reporting deficiencies, and quality problems were mainly reflected in the reporting registration and protocol, comprehensive search strategy and additional analysis. GRADE results elevated the quality of evidence to be low or very low overall.

**Conclusions:**

Probiotics may have a therapeutic effect on RA, based on the evidence provided by the SRs/MAs in this overview. Nevertheless, there is still a lack of conclusive evidence due to methodological limitations in the included research. To make trustworthy judgments regarding the efficacy of probiotics in the treatment of RA, more large-scale, high-quality randomized controlled trials are still required.

## 1 Introduction

Rheumatoid arthritis (RA) is an autoimmune systemic inflammatory disease involving multiple joints in the human body (1). Failure to effectively block disease progression can lead to cartilage and bone erosion, ultimately leading to joint deformity (2). RA is the most common inflammatory disease, with a global prevalence of 0.5 to 1% (3). Although many factors have been reported that may have a significant impact on the development and progression of RA, the pathophysiologic mechanisms of RA remain unelucidated (4). Accumulating evidence reveals an association between gut microbe and RA (5). In contrast to normal rats, germ-free rats are more likely to develop RA and have more severe symptoms (6). In addition, patients with inflammatory bowel disease who have disturbed gut microbe are also more likely to exhibit joint inflammation (7). Furthermore, fasting and vegan diets have been associated with reduced RA activity attributed to altered gut microbiota (8, 9). Therefore, modulation of gut microbes has been recognized as a potential strategy for the treatment of patients with RA (10).

Probiotics have recently demonstrated encouraging outcomes when used as adjuvant therapy for treating RA (11). Probiotics are described as “living microorganisms that, when ingested in sufficient quantities, provide a health benefit to the host” because they can decrease the number of harmful bacteria by competing for nutrition and colonization sites (12). The effectiveness of probiotics for RA has been assessed in a number of overlapping systematic reviews (SRs) and meta-analyses (MAs) to date (13–19). Nevertheless, there has been inconsistent evidence from these SRs/MAs. Trustworthy evidence is produced by high-quality SRs/MAs, while low-quality SRs and MAs may unintentionally affect choices (20, 21). As a result, when several studies with similar findings are published, an overview of prior SRs/MAs on the subject is frequently required (22). The purpose of this study was to offer evidence for clinical decisions by methodically gathering, assessing, and synthesizing prior SRs/MAs on the use of probiotics in the treatment of RA.

## 2 Methods

### 2.1 Included and excluded criteria

The following inclusion criteria were taken into consideration: (a) SRs/MAs that used probiotics for RA were eligible; (b) patients with a clear diagnosis of RA; (c) studies comparing probiotics to placebo were eligible; (d) disease activity score (DAS), swollen joints count (SJC), level of C-reactive protein (CRP) and tumor necrosis factor-α (TNF-α) were used as outcomes. The eligibility of each article was established by consensus between the two reviewers. The following exclusion criteria were taken into consideration: (a) studies unrelated to the subject; (b) conference proceedings and protocols; (c) animal experiments.

### 2.2 Strategy for searching

We comprehensively searched Embase, PubMed, Web of Science, Cochrane Library, and screened qualified SRs that had been released from database inception to February 12, 2024. We used a mix of free keywords and Mesh phrases to perform our search. The keywords mainly included RA, probiotics SA, and MA. The search strategy for PubMed is presented in **Table 1**, which was adjusted to adapt to different databases.

**Table 1:** Search strategy for PubMed.

Query	Search term
# 1	Rheumatoid arthritis [Mesh]
# 2	Rheumatoid arthritis [Title/Abstract] OR Arthritis rheumatoid [Title/Abstract] OR RA [Title/Abstract]
# 3	#1 OR #2
# 4	Probiotics [Mesh]
# 5	Probiotic [Title/Abstract] OR beneficial bacteria [Title/Abstract] OR microecological preparation [Title/Abstract] OR lactobacillus [Title/Abstract] OR streptococcus thermophilus [Title/Abstract] OR bifidobacterium [Title/Abstract] OR clostridium butyricum [Title/Abstract] OR saccharomyces [Title/Abstract] OR bacillus [Title/Abstract]
# 6	#4 OR #5
# 7	Meta-analysis as Topic [Mesh]
# 8	Systematic review [Title/Abstract] OR meta-analyses [Title/Abstract] OR meta analyses [Title/Abstract] OR meta-analysis OR metaanalysis [Title/Abstract]
# 9	#7 OR #8
# 8	#3 AND #6 AND #9

### 2.3 Data collection and extraction

Two reviewers independently conducted literature screening. All search results were imported into Endnote 20 to remove duplicates and inconsistent articles were removed based on the title and abstract. Finally, the full text was read out and eligible SRs were included. Two reviewers independently extracted the basic characteristics of eligible literature, including author, publication year, diagnostic criteria, sample size, intervention, comparison, and outcomes. Two reviewers crosschecked what was extracted, and consulted a third reviewer for any discrepancies.

### 2.4 Methodological evaluation

Using AMSTAR-2 tool (23), the methodological quality of the included SRs/MAs was evaluated independently by two investigators. A third investigator was consulted on any disagreement. Out of the 16 items that the tool evaluates, 7 are considered essential domains (items 2, 4, 7, 9, 11, 13, and 15). Three possibilities remain for the evaluation: “Yes,” “Partially Yes,” and “No.”

### 2.5 Reporting quality appraisal

Using PRISMA checklists (24), the reporting quality of the included SRs/MAs was evaluated independently by two investigators. A third investigator was consulted on any disagreement. Out of the 7 sections that the tool evaluates, 27 checklists are considered essential domains. Three possibilities remain for the evaluation: “Yes,” “Partially Yes,” and “No.”

### 2.6 Evidence quality evaluation

Using GRADE system (25), the evidence quality of the included SRs/MAs was evaluated independently by two investigators. A third investigator was consulted on any disagreement. The publication bias, bias risk, inconsistency, indirectness and imprecision are used to evaluate the GRADE scoring system. Four categories are used to group the results: high, moderate, low, and very low.

## 3 Results

### 3.1 Selection of literature

**Figure 1:**
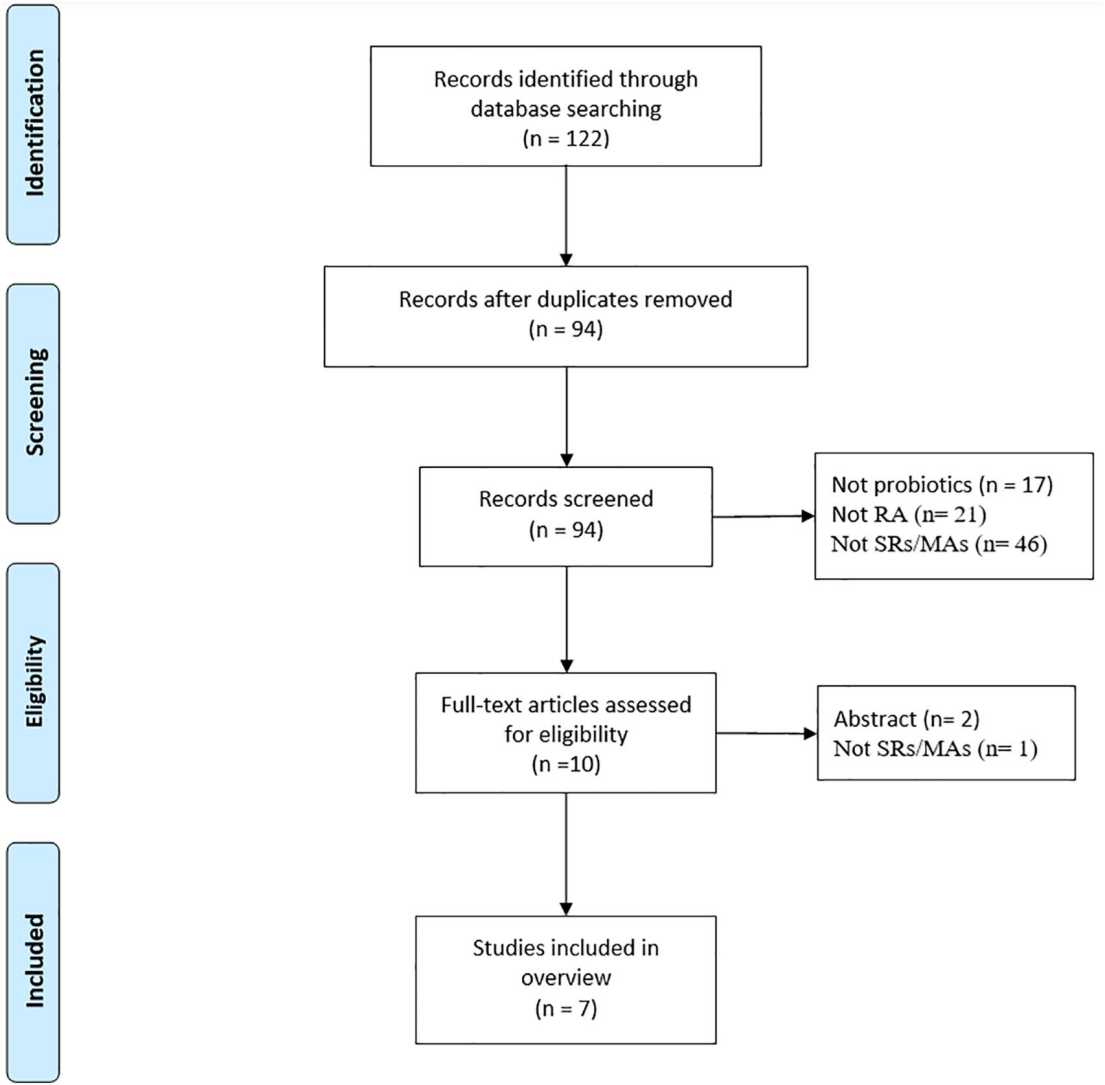
Publication selection procedure.

A total of 122 studies were retrieved, and 94 studies were obtained after excluding duplicate studies. After reading the titles and abstract, 10 literatures were obtained. Of the remaining studies, 3 studies were excluded: conference abstracts (n = 2), not SR/MA (n = 1). Ultimately, this review includes 12 SR-MAs (13–19) that satisfied the inclusion requirements. The literature screening process is illustrated in **Figure 1**.

### 3.2 Study characteristics

A summary of the data extracted from the seven SRs/MAs is provided in **Table 2**. These include the SRs/MAs published between 2017 and 2023. All reviews were published in English. The number of trails included in these SRs varied widely, ranging from 4 to 10, and the total number of participants ranged from 153 to 632. Interventions in the therapy group were probiotics, whereas placebo was used in the control group. Almost all SRs/MAs reached a positive conclusion.

**Table 2:** Characteristics of the included reviews.

Studies	Country	Trials (subjects)	Experimental Intervention	Control Intervention	Outcomes	Conclusion summary
Mohammed, 2017 (13)	Egypt	9 (361)	Probiotics	Placebo	①, ②, ③, ④	Probiotics lowered the pro-inflammatory cytokine IL-6, which is an indicator for joint destruction in RA; however, the clinical effect of probiotics is still unclear.
Pan, 2017 (14)	China	6 (249)	Probiotics	Placebo	①, ③, ④	We found that probiotic supplementation may show a limited improvement in RA therapy in existing reports because of a lack of sufficiently high-quality work on the part of clinicians.
Rudbane, 2018 (15)	Iran	4 (153)	Probiotics	Placebo	①, ②, ③, ④	Probiotics seem to be less effective in RA; however, to reach a firm conclusion, we need further evidence.
Lowe, 2020 (16)	Australia	8 (378)	Probiotics	Placebo	①, ③	This review indicates probiotics are generally safe to take for older individual with established RA alongside many common rheumatology medications, but they have not been investigated alongside the newer medications.
Sanchez, 2022 (17)	France	8 (344)	Probiotics	Placebo	①, ③	Probiotic supplementation might decrease RA activity with a moderate decrease effect on CRP, but lack of evidence and studies’ heterogeneity do not allow us to propose them to patients with inflammatory arthritis to control their disease.
Zeng, 2022 (18)	China	10 (632)	Probiotics	Placebo	①, ②, ③	Probiotic supplements may improve RA. However, more randomized controlled trials are needed in the future to determine the efficacy and optimal dosing design of probiotics.
Yuan, 2023 (19)	China	9 (385)	Probiotics	Placebo	①, ③, ④	The effects of probiotic consumption on RA are very beneficial and have some reference significance for formulating treatment guidelines for RA. However, more trials are needed to confirm the influence of probiotics on RA patients.

①: disease activity score; ②: swollen joints count; ③: level of C-reactive protein; ④: tumor necrosis factor-α (TNF-α).

### 3.3 Methodological evaluation

AMASTAR-2 was used to assess the methodological quality, two included studies were rated as moderate quality, and the remaining studies ware rated as critically low quality. The key factors affecting the quality of the studies included items 2 (only two reviews registered a protocol), 4 (only two reviews used a comprehensive literature search strategy), and 7 (no reviews provided a list of excluded studies and justified the exclusions). The detailed results are presented in **Table 3**.

**Table 3:** Quality assessment of the included reviews by the AMSTAR-2 tool.

Author, Year	AMSTAR-2																Quality
	Q1	Q2	Q3	Q4	Q5	Q6	Q7	Q8	Q9	Q10	Q11	Q12	Q13	Q14	Q15	Q16	
Mohammed, 2017 (13)	Y	Y	Y	Y	Y	Y	N	Y	Y	Y	Y	Y	Y	Y	Y	Y	Moderate
Pan, 2017 (14)	Y	N	Y	PY	Y	Y	N	Y	Y	Y	Y	Y	Y	Y	Y	Y	Very low
Rudbane, 2018 (15)	Y	N	Y	Y	Y	Y	N	Y	Y	Y	Y	Y	Y	Y	Y	Y	Very low
Lowe, 2020 (16)	Y	Y	Y	Y	Y	Y	N	Y	Y	Y	Y	Y	Y	Y	Y	Y	Moderate
Sanchez, 2022 (17)	Y	N	Y	Y	Y	Y	N	Y	Y	Y	Y	Y	Y	Y	Y	Y	Very low
Zeng, 2022 (18)	Y	N	Y	PY	Y	Y	N	Y	Y	Y	Y	Y	Y	Y	Y	Y	Very low
Yuan, 2023 (19)	Y	N	Y	Y	Y	Y	N	Y	Y	Y	Y	Y	Y	Y	Y	Y	Very low

Y, yes; PY, partial yes; N, no.

### 3.4 Reporting quality appraisal

PRISMA was used to assess the reporting quality, and overall, the quality of reporting remains not fully satisfactory. The key factors affecting the quality of the studies included Q5 (only two reviews registered a protocol), Q8 (only two reviews used a comprehensive literature search strategy), Q16 (three reviews provided an additional analysis), and Q23 (three reviews provided an additional analysis). The detailed results are presented in **Table 4**.

**Table 4:** Results of the reporting quality.

Section/topic	Items	Mohammed, 2017 (13)	Pan, 2017 (14)	Rudbane, 2018 (15)	Lowe, 2020 (16)	Sanchez, 2022 (17)	Zeng, 2022 (18)	Yuan, 2023 (19)	Compliance (%)
Title	Q1. Title	Y	Y	Y	Y	Y	Y	Y	100%
Abstract	Q2. Structured summary	Y	Y	Y	Y	Y	Y	Y	100%
Introduction	Q3. Rationale	Y	Y	Y	Y	Y	Y	Y	100%
	Q4. Objectives	Y	Y	Y	Y	Y	Y	Y	100%
Methods	Q5. Protocol and registration	Y	N	N	Y	N	N	N	28.57%
	Q6. Eligibility criteria	Y	Y	Y	Y	Y	Y	Y	100%
	Q7. Information sources	Y	Y	Y	Y	Y	Y	Y	100%
	Q8. Search	Y	PY	Y	Y	Y	PY	Y	71.43%
	Q9. Study selection	Y	Y	Y	Y	Y	Y	Y	100%
	Q10. Data collection process	Y	Y	Y	Y	Y	Y	Y	100%
	Q11. Data items	Y	Y	Y	Y	Y	Y	Y	100%
	Q12. Risk of bias in individual studies	Y	Y	Y	Y	Y	Y	Y	100%
	Q13. Summary measures	Y	Y	Y	Y	Y	Y	Y	100%
	Q14. Synthesis of results	Y	Y	Y	Y	Y	Y	Y	100%
	Q15. Risk of bias across studies	Y	Y	Y	Y	Y	Y	Y	100%
	Q16. Additional analyses	N	Y	N	Y	Y	N	Y	57.14%
Results	Q17. Study selection	Y	Y	Y	Y	Y	Y	Y	100%
	Q18. Study characteristics	Y	Y	Y	Y	Y	Y	Y	100%
	Q19. Risk of bias within studies	Y	Y	Y	Y	Y	Y	Y	100%
	Q20. Results of individual studies	Y	Y	Y	Y	Y	Y	Y	100%
	Q21. Synthesis of results	Y	Y	Y	Y	Y	Y	Y	100%
	Q22. Risk of bias across studies	Y	Y	Y	Y	Y	Y	Y	100%
	Q23. Additional analysis	N	Y	N	Y	Y	N	Y	57.14%
Discussion	Q24. Summary of evidence	Y	Y	Y	Y	Y	Y	Y	100%
	Q25. Limitations	Y	Y	Y	Y	Y	Y	Y	100%
	Q26. Conclusions	Y	Y	Y	Y	Y	Y	Y	100%
Funding	Q27. Funding	Y	Y	Y	Y	Y	Y	Y	100%

Y, yes; PY, partial yes; N, no.

### 3.5 Evidence quality evaluation

The seven SRs included 21 outcomes related to the treatment of RA with probiotics. The results showed that 16 (19.05%) were rated as low quality and 5 (80.95%) as critically low quality. The risk of bias (21/21, 100%), imprecision (21/21,100%) and inconsistency (15/21,71.43%) were the main factors in obtaining the results. The details are presented in **Table 5**.

**Table 5:** Results of Evidence Quality.

Review	Outcomes	№ of trails	Certainty assessment					№ of patients		Relative effect (95% CI)	Quality
			Limitations	Inconsistency	Indirectness	Imprecision	Publication bias	Experimental	Control		
Mohammed, 2017 (13)	DAS	3	Serious^a^	No	No	Serious^c^	No	67	65	MD 0.023 [-0.584, 0.631]	⨁⨁⨁◯◯ Low
	SJC	5	Serious^a^	No	No	Serious^c^	No	96	95	MD 0.171 [-0.391, 0.733]	⨁⨁⨁◯◯ Low
	CRP	5	Serious^b^	Serious^b^	No	Serious^c^	No	96	95	MD -2.660 [-6.144, 0.823]	⨁⨁◯◯◯ Very low
	TNF-α	4	Serious^a^	Serious^b^	No	Serious^c^	No	64	63	MD -0.092 [-0.940, 0.756]	⨁⨁◯◯◯ Very low
Pan, 2017 (14)	DAS	3	Serious^a^	Serious^b^	No	Serious^c^	No	67	65	MD -0.11 [-0.47, 0.24]	⨁⨁◯◯◯ Very low
	CRP	4	Serious^a^	Serious^b^	No	Serious^c^	No	75	78	MD -0.77 [-1.48, -0.05]	⨁⨁◯◯◯ Very low
	TNF-α	2	Serious^a^	No	No	Serious^c^	No	59	59	MD -1.35 [-1.99, -0.71]	⨁⨁⨁◯◯ Low
Rudbane, 2018 (15)	DAS	2	Serious^a^	No	No	Serious^c^	No	53	53	MD -0.58 [-0.97, -0.19]	⨁⨁⨁◯◯ Low
	SJC	4	Serious^a^	No	No	Serious^c^	No	76	78	SMD -0.30 [-0.62, 0.02]	⨁⨁⨁◯◯ Low
	CRP	4	Serious^a^	Serious^b^	No	Serious^c^	No	66	66	SMD -0.32 [-0.66, 0.00]	⨁⨁⨁◯◯ Low
	TNF-α	3	Serious^a^	Serious^b^	No	Serious^c^	No	59	59	SMD 0.01 [-1.41, 1.43]	⨁⨁⨁◯◯ Low
Lowe, 2020 (16)	DAS	4	Serious^a^	Serious^b^	No	Serious^c^	No	105	105	MD-0.28 [-0.50, -0.05]	⨁⨁⨁◯◯ Low
	CRP	7	Serious^a^	Serious^b^	No	Serious^c^	No	162	163	MD -2.34 [-4.26, -0.41]	⨁⨁⨁◯◯ Low
Sanchez, 2022 (17)	DAS	3	Serious^a^	Serious^b^	No	Serious^c^	No	72	68	SMD -0.54 [-1.94, 0.85]	⨁⨁◯◯◯ Very low
	CRP	5	Serious^a^	Serious^b^	No	Serious^c^	No	93	89	MD -3.04 [-4.47, -1.62]	⨁⨁⨁◯◯ Low
Zeng, 2022 (18)	DAS	4	Serious^a^	Serious^b^	No	Serious^c^	No	122	121	MD -0.55 [-1.33, 0.24]	⨁⨁⨁◯◯ Low
	SJC	4	Serious^a^	Serious^b^	No	Serious^c^	No	103	107	SMD -0.10 [-0.64, 0.44]	⨁⨁⨁◯◯ Low
	CRP	5	Serious^a^	Serious^b^	No	Serious^c^	No	385	374	SMD -1.57 [-2.98, -0.15]	⨁⨁⨁◯◯ Low
Yuan, 2023 (19)	DAS	6	Serious^a^	Serious^b^	No	Serious^c^	No	135	136	MD -0.39 [-0.61, -0.17]	⨁⨁⨁◯◯ Low
	CRP	6	Serious^a^	Serious^b^	No	Serious^c^	No	124	128	MD -2.01 [-3.23, -0.80]	⨁⨁⨁◯◯ Low
	TNF-α	5	Serious^a^	No	No	Serious^c^	No	87	87	MD -1.03 [-1.40, -0.67]	⨁⨁⨁◯◯ Low

IBS-SSS, IBS symptom severity scale; QoL, quality of life. a: the experimental design had a large bias in random, distributive findings or was blind; b: the confidence interval overlaps less, the heterogeneity test P was very small, and the I^2^ was larger; c: the Confidence interval was not narrow enough, or the simple size is too small; d: funnel graph asymmetry, or fewer studies were included and there may have been greater publication bias.

## 4 Discussion

The level of evidence from SRs is considered to be the highest (26, 27), provided that the SRs stand up to the process of producing evidence.

### 4.1 A definitive conclusion cannot be reached

In the seven included articles, all literatures reported positive results, which indicated the prospect of treatment of RA with probiotics. However, due to the low methodological quality and evidence quality of the included SRs/MAs, the results of these studies should be treated with caution. At present, due to factors such as the pathogenesis of RA and the lack of objective diagnostic criteria for RA, the long-term clinical efficacy evaluation of RA is not satisfactory, and more studies are needed in the future to conduct in-depth analysis and discussion on the above issues. In addition, the existing clinical studies also have shortcomings, such as small sample size, short follow-up time, and inconsistent efficacy evaluation. There is still insufficient evidence to confirm the superiority of probiotics treatment for RA. In the future, it is necessary to improve the quality of the original trails and conduct more prospective clinical studies with large samples and multiple centers to provide more high-quality evidence-based evidence for clinical workers to apply probiotics treatment for RA.

### 4.2 Research deficiencies to be improved

No SR was rated as high quality according to AMSTAR-2 tool, and no SR reported all 27 entries according to PRISMA. The risk of bias was high of the included SRs for the following reasons: no comprehensive retrieval strategy was adopted, failure to provide a list of excluded documents with reasons, evaluation and discussion of publication bias and evidence credibility, insufficient analysis of sources of heterogeneity and their impact on results, and lack of reporting and discussion of registration options and funding sources. According to the Cochrane handbook, all systematic evaluations should report the registered protocol and registration number in the methodology section (28). Unfortunately, none of the included studies declared that they had registered the protocol and provided the registration number, so we determined that they did not have a prior registered protocol, which increases the risk of bias while decreasing the transparency of the study (29). In addition, additional analyses, such as subgroup analysis, sensitivity analysis, and publication bias assessment, were not performed in the included studies when performing MA, which means that the efficacy of different probiotics for different populations of RA has not been precisely explored, and the results of current SRs are not necessarily robust and are likely to be altered by further analysis (30). According to GRADE evaluation results, all included outcomes were low or extremely low, indicating that the conclusions of the included SRs/MAs are likely to be significantly different from the actual situation. The main factors contributing to the evidence downgrade were risk of bias, imprecision and inconsistency. Almost all the original SR/MA-included trials had some defects in randomization, hiding blindness, and follow-up, and the risk of publication bias was high. Small sample sizes trails with a lack of randomization, blinding, and allocation concealment cause evidence quality to range from moderate to very low. To guarantee the availability of evidence, future SRs/MAs must be planned and carried out strictly in accordance with AMSTAR-2 and PRISMA.

### 4.3 Strengths and limitations

Insofar as we are aware, this study offers the first thorough evaluation and synopsis of the evidence bolstering probiotic usage in RA. But there are some limitations that need to be recognized. First, the outcomes of various original studies included in the relevant SR/MA are different, and the number of original studies and sample size of some outcome indicators are small, which can lead to low evidence strength of outcome indicators. Furthermore, due to the large difference in the outcome indicators of the included studies, quantitative combination and analysis were not carried out in this study, and only the study was described according to the conclusion of the original text.

## 5 Conclusion

Probiotics may have a therapeutic effect on RA, based on the evidence provided by the SRs/MAs in this overview. Nevertheless, there is still a lack of conclusive evidence due to methodological limitations in the included research. To make trustworthy judgments regarding the efficacy of probiotics in the treatment of RA, more large-scale, high-quality randomized controlled trials are still required.

## Data availability statement

The original contributions presented in the study are included in the article/supplementary material. Further inquiries can be directed to the corresponding author.

## Author contributions

WL: Conceptualization, Writing – original draft. YZ: Conceptualization, Data curation, Writing – original draft. DG: Conceptualization, Methodology, Writing – original draft. RG: Conceptualization, Data curation, Writing – original draft. JY: Conceptualization, Data curation, Writing – original draft. HY: Writing – original draft, Writing – review & editing.

## References

[1] Smith MH Berman JR . What is rheumatoid arthritis? JAMA. (2022) 327:1194. doi: 10.1001/jama.2022.0786 35315883

[2] Sayah A English JC 3rd . Rheumatoid arthritis: a review of the cutaneous manifestations. J Am Acad Dermatol. (2005) 53:191–209; quiz 210–2. doi: 10.1016/j.jaad.2004.07.023 16021111

[3] Lopez-Corbeto M Martínez-Mateu S Pluma A Ferrer R López-Lasanta M De Agustín JJ . The ovarian reserve as measured by the anti-Müllerian hormone is not diminished in patients with rheumatoid arthritis compared to the healthy population. Clin Exp Rheumatol. (2021) 39:337–43. doi: 10.55563/clinexprheumatol/73txen 32896242

[4] Zhao J Guo S Schrodi SJ He D . Molecular and cellular heterogeneity in rheumatoid arthritis: mechanisms and clinical implications. Front Immunol. (2021) 12:790122. doi: 10.3389/fimmu.2021.790122 34899757 PMC8660630

[5] Bergot AS Giri R Thomas R . The microbiome and rheumatoid arthritis. Best Pract Res Clin Rheumatol. (2019) 33:101497. doi: 10.1016/j.berh.2020.101497 32199713

[6] Scher JU Abramson SB . The microbiome and rheumatoid arthritis. Nat Rev Rheumatol. (2011) 7:569–78. doi: 10.1038/nrrheum.2011.121 PMC3275101 21862983

[7] Saarela M Lähteenmäki L Crittenden R Salminen S Mattila-Sandholm T . Gut bacteria and health foods–the European perspective. Int J Food Microbiol. (2002) 78:99–117. doi: 10.1016/S0168-1605(02)00235-0 12222640

[8] Peltonen R Nenonen M Helve T Hänninen O Toivanen P Eerola E . Faecal microbial flora and disease activity in rheumatoid arthritis during a vegan diet. Br J Rheumatol. (1997) 36:64–8. doi: 10.1093/rheumatology/36.1.64 9117178

[9] Müller H de Toledo FW Resch KL . Fasting followed by vegetarian diet in patients with rheumatoid arthritis: a systematic review. Scand J Rheumatol. (2001) 30:1–10. 11252685 10.1080/030097401750065256

[10] Zhao T Wei Y Zhu Y Xie Z Hai Q Li Z . Gut microbiota and rheumatoid arthritis: From pathogenesis to novel therapeutic opportunities. Front Immunol. (2022) 13:1007165. doi: 10.3389/fimmu.2022.1007165 36159786 PMC9499173

[11] Bungau SG Behl T Singh A Sehgal A Singh S Chigurupati S . Targeting probiotics in rheumatoid arthritis. Nutrients. (2021) 13:3376. doi: 10.3390/nu13103376 34684377 PMC8539185

[12] Ferro M Charneca S Dourado E Guerreiro CS Fonseca JE . Probiotic supplementation for rheumatoid arthritis: A promising adjuvant therapy in the gut microbiome era. Front Pharmacol. (2021) 12:711788. doi: 10.3389/fphar.2021.711788 34366867 PMC8346200

[13] Mohammed AT Khattab M Ahmed AM Turk T Sakr N M Khalil A . The therapeutic effect of probiotics on rheumatoid arthritis: a systematic review and meta-analysis of randomized control trials. Clin Rheumatol. (2017) 36:2697–707. doi: 10.1007/s10067-017-3814-3 28914373

[14] Pan H Li R Li T Wang J Liu L . Whether probiotic supplementation benefits rheumatoid arthritis patients: A systematic review and meta-analysis. Engineering. (2017) 3:115–21. doi: 10.1016/J.ENG.2017.01.006

[15] Aqaeinezhad Rudbane SM Rahmdel S Abdollahzadeh SM Zare M Bazrafshan A Mazloomi SM . The efficacy of probiotic supplementation in rheumatoid arthritis: a meta-analysis of randomized, controlled trials. Inflammopharmacology. (2018) 26:67–76. doi: 10.1007/s10787-017-0436-y 29302905

[16] Lowe JR Briggs AM Whittle S Stephenson MD . A systematic review of the effects of probiotic administration in inflammatory arthritis. Complement Ther Clin Pract. (2020) 40:101207. doi: 10.1016/j.ctcp.2020.101207 32771911

[17] Sanchez P Letarouilly JG Nguyen Y Sigaux J Barnetche T Czernichow S . Efficacy of probiotics in rheumatoid arthritis and spondyloarthritis: A systematic review and meta-analysis of randomized controlled trials. Nutrients. (2022) 14:354. doi: 10.3390/nu14020354 35057535 PMC8779560

[18] Zeng L Deng Y He Q Yang K Li J Xiang W . Safety and efficacy of probiotic supplementation in 8 types of inflammatory arthritis: A systematic review and meta-analysis of 34 randomized controlled trials. Front Immunol. (2022) 13:961325. doi: 10.3389/fimmu.2022.961325 36217542 PMC9547048

[19] Yuan Y Ji W Lin Z Gan K . Benefits of probiotics in rheumatoid arthritis patients: A systematic review and meta-analysis. Trop J Pharm Res. (2023) 22:399–406. doi: 10.4314/tjpr.v22i2.24

[20] Huang J Zhang J Wang Y Ma J Yang X Guo X . Scientific evidence of chinese herbal medicine (Gegen qinlian decoction) in the treatment of ulcerative colitis. Gastroenterol Res Pract. (2022) 2022:7942845. doi: 10.1155/2022/7942845 35356743 PMC8958105

[21] Huang J Lu M Zheng Y Ma J Ma X Wang Y . Quality of evidence supporting the role of acupuncture for the treatment of irritable bowel syndrome. Pain Res Manag. (2021) 2021:2752246. doi: 10.1155/2021/2752246 34956431 PMC8694972

[22] Saunders H Gallagher-Ford L Kvist T Vehviläinen-Julkunen K . Practicing healthcare professionals' Evidence-based practice competencies: an overview of systematic reviews. Worldviews Evid Based Nurs. (2019) 16:176–85. doi: 10.1111/wvn.12363 31074582

[23] Shea BJ Reeves BC Wells G Thuku M Hamel C Moran J . AMSTAR 2: a critical appraisal tool for systematic reviews that include randomised or non-randomised studies of healthcare interventions, or both. BMJ. (2017) 358:j4008. doi: 10.1136/bmj.j4008 28935701 PMC5833365

[24] Liberati A Altman DG Tetzlaff J Mulrow C Gøtzsche PC Ioannidis JP . The PRISMA statement for reporting systematic reviews and meta-analyses of studies that evaluate health care interventions: explanation and elaboration. PloS Med. (2009) 6:e1000100. doi: 10.1371/journal.pmed.1000100 19621070 PMC2707010

[25] Guyatt GH Oxman AD Vist GE Kunz R Falck-Ytter Y Alonso-Coello P . GRADE: an emerging consensus on rating quality of evidence and strength of recommendations. BMJ. (2008) 336:924–6. doi: 10.1136/bmj.39489.470347.AD PMC2335261 18436948

[26] Yang K Zhang J Zhao L Cheng L Li Y Kang Y . An umbrella review of Lianhua Qingwen combined with Western medicine for the treatment of coronavirus disease 2019. Acupuncture Herbal Med. (2022) 2:143−151. doi: 10.1097/HM9.0000000000000041 PMC9746252 37808351

[27] Chen Z Jiang T Peng Y Qiang X Yang F Hu H . Acupuncture and moxibustion treating lower urinary tract symptoms due to benign prostatic hyperplasia: a systematic review and network meta-analysis. Acupuncture Herbal Med. (2022) 2:84−90. doi: 10.1097/HM9.0000000000000029

[28] Cumpston M Li T Page MJ Chandler J Welch VA Higgins JP . Updated guidance for trusted systematic reviews: a new edition of the Cochrane Handbook for Systematic Reviews of Interventions. Cochrane Database Syst Rev. (2019) 10:ED000142. doi: 10.1002/14651858 31643080 PMC10284251

[29] Huang J Liu H Chen J Cai X Huang Y . The effectiveness of tai chi in patients with breast cancer: an overview of systematic reviews and meta-analyses. J Pain Symptom Manage. (2021) 61:1052–9. doi: 10.1016/j.jpainsymman.2020.10.007 33068706

[30] Huang J Shen M Qin X Guo W Li H . Acupuncture for the treatment of tension-type headache: an overview of systematic reviews. Evid Based Complement Alternat Med. (2020) 2020:4262910. doi: 10.1155/2020/4262910 32256645 PMC7106880

